# Financial Stock Investment Management Using Deep Learning Algorithm in the Internet of Things

**DOI:** 10.1155/2022/4514300

**Published:** 2022-07-16

**Authors:** Jianjuan Fan, Shen Peng

**Affiliations:** ^1^Faculty of Information, University of Toronto, Ontario, Canada; ^2^Computer Science and Technology, Guangzhou University, Guangzhou 510006, China

## Abstract

This paper aims to explore a new model to study financial stock investment management (SIM) and obtain excess returns. Consequently, it proposes a financial SIM model using deep Q network (DQN) as reinforcement earning (RL) algorithm and Long Short-Term Memory (LSTM) as deep neural network (DNN). Then, after training and optimization, the proposed model is back-tested. The research findings are as follows: the LSTM neural network (NN)-based model will import the observation of the market at each time and the change of transaction information over time. The LSTM network can find and learn the potential relationship between time series data. There are two hidden layers and one output layer in the model. The hidden layer is an LSTM structure and the output layer is the fully connected NN. DQN algorithm first stores the experience sample data of the agent-environment interaction into the experience pool. It then randomly selects a small batch of data from the experience pool to train the network. Doing so removes the correlation and dependence between samples so that the DNN model can better learn the value function in the RL task. The model can predict the future state according to historical information and decide which actions to take in the next step. Meanwhile, five stocks of Chinese A-shares are selected to form an asset pool. The initial 500,000 amount of the account is divided into five equal shares, which are invested and traded. Overall, the model account's rate of return (RoR) during the back-test is 32.12%. The Shanghai Stock Exchange (SSI) has risen by 19.157% in the same period. Thus, the model's performance has exceeded the SSI's in the same period. E stock has the maximum RoR of 78.984%. The RoR of A, B, and C stocks is 54.129%, 11.594%, and 9.815%, respectively. B stock presents a minimum RoR of 6.084%. All these stocks have got positive returns. Therefore, the proposed financial SIM based on the DL algorithm is scientific and feasible. The research content has certain significant reference for the DL-based financial SIM.

## 1. Introduction

The rapid domestic economic development provides venture capitalists (VCs) with huge profit space in the stock market. The stock market is paid more and more attention to by the people, including the academic, industrial, and individual VCs [1]. However, due to excessive numbers of individual VCs and serious irrational investments in the domestic market, the stock price rises or falls sharply, making the market cycle complex and unstable. Meanwhile, with the increasing expansion of available data, it is difficult to completely extract valuable information from complex data by human analysis alone [2]. Therefore, how to reasonably and effectively manage the investment of financial stocks has become a problem studied by more scholars worldwide. With the continuous expansion of data and the upgrading and renewal of computer software and hardware infrastructure, deep learning (DL) has achieved blowout development in the era of big data. The financial field usually contains a large amount of data. High dimensions and high noise characterize these data. The traditional econometric methods are sometimes not suitable for analyzing such data. Financial data prediction has always been a challenging problem in the financial field [3].

The DL can tackle the complex high-dimensional financial data and lend itself to learning control strategies through data representation. Therefore, the DL algorithm provides a new idea for financial stock investment management (SIM). To cite a few examples, Guo et al. [4] tried to build illiquidity and idiosyncratic volatility indexes. After controlling idiosyncratic volatility indexes, they found a significant positive correlation between illiquidity indexes and equity risk premium. Arnott and Harvey [5] demonstrated the principles and procedures of applying machine learning (ML) in the financial field from the dimensions of research motivation, statistical methods, sample data selection, model complexity, and model cross-validation. Simonian and Wu [6] used a ridge regression model to replicate hedge funds and avoid model overfitting through the objective function (OF) regularization. The present work proposes a method to select the best regularization term coefficient for a time series. The empirical results show that the ridge regression model outperforms the ordinary least squares (OLS) regression model over some time. Additionally, more investment strategies based on DL algorithms have come into the public view in recent years. However, most current DL-based trading strategies focus on portfolio screening and rise and fall prediction. There is still less research on how to reduce trading risk effectively. Based on the traditional single objective reinforcement learning (RL), this paper creatively puts forward a multiobjective deep reinforcement learning (DRL) model. It aims to reduce transaction risks and volatility while maximizing the total return of the account.

This paper intends to explore new models to study financial SIM and reduce stock volatility while obtaining excess returns. This study applies the deep Q network (DQN) algorithm to the financial SIM. Firstly, the Long Short-Term Memory (LSTM) neural network (NN) is studied, and the model based on LSTM is implemented. The transaction action is transformed to the specified value range with a one-layer fully connected network. Then, the DQN algorithm is used to train the model. The training results are normalized. Meanwhile, the gradient descent method (GDM) is used to optimize the model. Finally, the trained model is back-tested to verify the training results and prove the scientificity and feasibility. This research can improve the return and reduce the transaction risk through the multiobjective DRL model. It has a certain reference significance for the financial management based on the DL algorithm.

## 2. Financial SIM Based on DL

In the traditional investment theory, the investment method of stock market research is usually nonquantitative, and the most common classification is the fundamental analysis (FA) and technical analysis (TA) method [7]. The FA method analyzes a country, an industry, and a specific company from the perspective of politics, macroeconomy, and microeconomy. It judges whether the macroeconomic environment, interindustry competition, intraindustry competition, and corporate governance level can improve the stock price, thereby helping VCs decide whether to invest in the stock. VCs need to study the company's fundamentals, financial statements, and financial ratios before investing. The FA method uses income, growth expectation, RoR, net growth rate (NGR), and other macro data to evaluate the enterprise's current financial status and growth potential. It also quantifies the “internal value” of the enterprise and judges whether it is suitable for long-term investment [8, 9]. VCs who use FA believe that the stock price is mainly affected by enterprise-level, national-level, or even international-level political and economic factors. Studying the supply-demand curve, macroeconomic, psychological, and policy factors can predict the development trend of the financial market (FM). Product innovation has also been developed along with other indexes, such as financial ratio, management policy, and marketing strategy, to help study the fundamentals of the company. Meanwhile, VCs also collect information, such as the enterprise's financial report, industry dynamics, and market trends, to accurately analyze enterprises' basic value and growth potential [10, 11].

The premise of TA is that the historical data of stocks reflect the future stock prices [12]. TA extracts the enterprise's basic situation and holds that the price is the manifestation of all the fundamental FM dynamics factors. TA does not explicitly consider the enterprise's internal and external features when studying market price changes, thus avoiding analyzing subjective economic factors. Besides, TA strives to identify the market price fluctuation laws and contends that the market transaction data are sufficient to determine the price trend. The view—history will reappear—helps researchers model the historical financial assets into time series and convert the transaction data into stock indexes mathematically, such as moving average (MA), relative strength index (RSI), momentum (MOM), and exponential moving average (EMA). Then, they draw relevant charts and analyze the price changes of the FM [13, 14]. Additionally, TA inputs historical prices and technical indexes, thus simplifying market trend prediction into pattern recognition and output the price dynamics, being a preferred short-term investment analysis approach. Lastly, TA processes transaction data utilizing mathematical tools, thus being more flexible than the FA that considers various factors [15].

**Figure 1:**
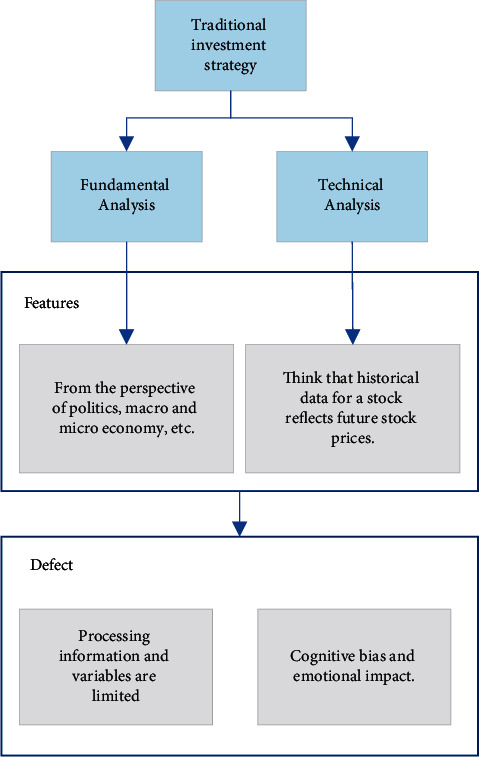
Traditional investment strategy.

Although the traditional investment strategy has gained some achievements, it still has some defects. First of all, the traditional active investment analyzes and forecasts the FM based on VCs' subjective judgment to generate artificial strategies, which is partly limited by the human brain's information processing ability, and thus, only a few variables are considered. Secondly, behavioral finance points out that VCs' psychology is often characterized by overconfidence and risk underestimation as well as blind conformity or regret. Cognitive bias and emotionalization are easy to harm investment decision-making (DM) [16–18]. The relationship between traditional investment strategies is shown in Figure 1.

## 3. LSTM NN

**Figure 2:**
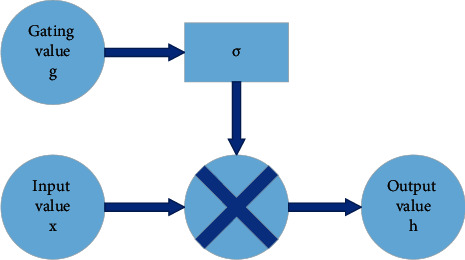
LSTM NN gating mechanism.

The concept of LSTM NN was first proposed in 1997. Unlike the basic RNN, the LSTM NN has stronger memorization, can express the implicit relationship between sequences faster, more accurately, and better, and is better at processing long sequence signal data. Since its proposal, LSTM has been extensively applied to predict sequences and natural language processing (NLP) in place of the basic RNN model [19]. Furthermore, the LSTM NN introduces two variables: the output and the memory, along with a gating mechanism to control the information flow. When the information is input into the LSTM NN, the information that meets the requirements is left, and the information that does not meet the criteria is forgotten [20, 21]. The gating mechanism of LSTM NN is depicted in Figure 2.

**Figure 3:**
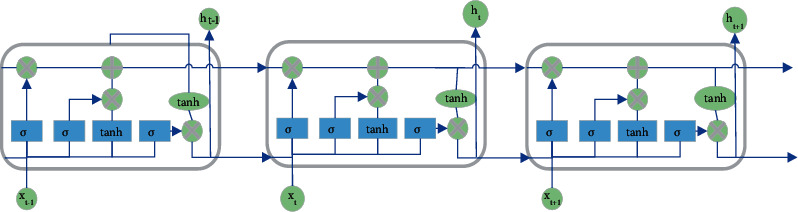
LSTM NN structure.

The LSTM NN has two state vectors: *C* and *h*, in which *C* is the internal state vector or the memory state vector and *h* represents the output vector.  Figure 3 projects the LSTM NN structure.

In Figure 3, the output vector *h*_*t*−1_ of the previous timestamp and the input *x*_*t*_ of the current timestamp pass through the AF tanh to obtain a new output vector *h*_*t*_. Compared with the basic RNN, LSTM only has one output vector (*h*) and adds a state vector *C*_*t*_. At the same time, the gating unit is used to control the forgetting and refreshing of information. The AF tanh is calculated by the following equation:

(1) $$\begin{array}{c} f{\left(x\right)}_{\text{tanh}}=\frac{e^{x}-e^{-x}}{e^{x}+e^{-x}}. \end{array}$$

**Figure 4:**
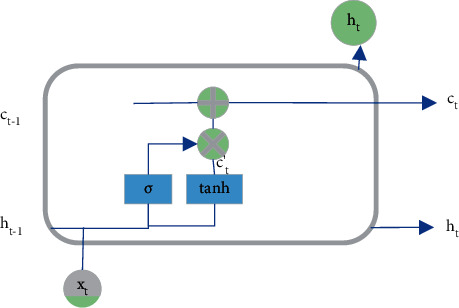
Schematic diagram of the input gate.

The input gate can control the LSTM NN input. Firstly, the input *x* at the current time and the output *h*_*t*−1_ at the previous time are transformed nonlinearly. The input vector is normalized to the interval of −1 and 1 through tanh AF. Yet, the input vector will not all be memorized by the LSTM NN but is controlled by the input gate. The control vector of the input gate comes from the nonlinear transformation of the input *x*_*t*_ and the output *h*_*t*−1_ of the previous timestamp through the AF, generally a sigmoid function [22]. The input gate control vector determines the acceptance degree of the LSTM NN to the new input vector of the current timestamp. When the control vector is 0, the LSTM NN does not accept any new input. In comparison, all new inputs are accepted when the control vector is 1. Controlled by the forget gate and the input gate, the LSTM NN selectively reads the memory of the previous timestamp and the new input of the current timestamp, forming a certain combination of these state vectors and, thereby determining the current timestamp's state vector.  Figure 4 shows the specific process.

**Figure 5:**
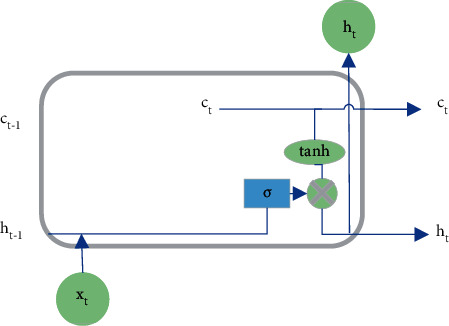
Schematic diagram of the output gate.

Unlike basic RNN, LSTM NN will not directly output the internal state vector. The state vector *h* of the basic RNN is both memory and output, so the state vector c equals the output vector *h*. By contrast, in the LSTM NN, the output gate controls the state vectors to be output. The output gate vector results from the offline combination of the state vector *h*_*t*−1_ of the previous timestamp and the input vector *x*_*t*_ through the AF transformation, generally a sigmoid function. When the output gate vector is 0, the output is closed, and the internal memory of the LSTM NN is completely blocked and cannot be used as the output. At this time, the vector 0 is output. Then, when the output gate vector is 1, the output is fully opened, and all the state vectors of the LSTM NN are used for output. The output of the LSTM NN comes from the multiplication of the output gate vector and the nonlinear transformation result of the state vector *c*_*t*_ through the tanh AF. In other words, the memory vector acts with the output gate after tanh AF to obtain the output of LSTM. Since the output gate vector falls into 0 and 1 and the tanh (*c*_*t*_) falls into −1 and 1, then the output of the LSTM NN falls into −1 and 1 [23].  Figure 5 specifies the process of LSTM output.

**Figure 6:**
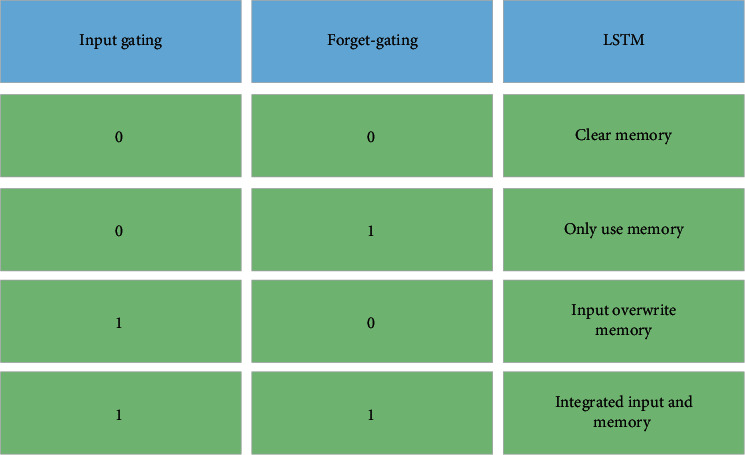
Gate behavior of the LSTM NN.

There are many state vectors and gate vectors in LSTM NNs, several nonlinear AFs are also used internally, and the calculation process is relatively complex. However, the function of each gate control is clear, and the typical gate control behavior is illuminated in Figure 6.

## 4. Financial SIM Model Based on DL

For the whole transaction model, the network structure of the model is essential because it determines the effect and convergence speed of model training. According to the time-series features of financial data, this paper hopes to design the model with two functionalities: one is to predict the future state according to historical information, and the other is to decide what action to take. When the LSTM NN is used to implement the financial SIM model, the input includes the observation of the market at each time and the change of transaction information over time. The LSTM NN can find and learn the potential relationship between time series data (TSD). Moreover, given the hardware conditions of the machine used in the training model, if the search space is too large, the multilayer dense NN may be too bulky to train, while the structure and weight sharing features of the LSTM NN reduce the difficulty of training [24, 25]. Accordingly, the proposed financial SIM model is based on the LSTM NN, including two hidden layers and a single output layer. Specifically, the hidden layer is the LSTM and the output layer is the fully connected NN.

Meanwhile, total return should be maximized while the trading strategy needs to consider the trading risk (mainly, the standard deviation (SD) of return). Therefore, to realize multiobjective trading strategy learning, this section first sets certain weights for different objectives and linearly fuses all objectives according to the set weights. Then, based on this objective training model, the investment strategy is finally output.

Subsequently, the SD of daily return is used to measure the fluctuation of daily return. The first OF of the trading model is the average daily return *R*, and the return at the current time is equal to the stock price variation-induced capital gain minus the transaction handling fee [26]. The second OF is the SD of average daily return *σ*. Then, based on the VC utility function *U* = *E*(*r*) − 0.5 *Aσ*^2^, the two OFs are linearly combined to obtain the total OF (U). Equations (2)–(4) calculate the multi-OF.

(2) $$\begin{array}{c} R=\frac{\left(1-c\right)\left(\sum_{t=1}^{T}R_{t}\sum_{i=1}^{n}R_{i}|\theta \right)}{T}, \end{array}$$

(3) $$\begin{array}{c} \sigma =\text{std}\left(R_{i}|\theta \right),\;i=1,2\ldots T, \end{array}$$

(4) $$\begin{array}{c} U=R-A\sigma^{2}. \end{array}$$

In equations (2)–(4), *T* indicates the model's accumulative transaction days, *n* signifies the single-day transactions, *θ* is the model parameter, and *c* denotes the transaction fee ratio, including stamp duty. Meanwhile, *A* means the weight corresponding to the fluctuation OF, and the total OF can be adjusted through the weight. Here, *A* is set to 0.2. Because the *U* calculated by the above equations is large, the network input cannot be ideal. Therefore, the total OF should be normalized. Simply put, the mean of all *U* is subtracted from each U, then divided by the SD of U, and finally, input into model training [27–29].

The action space of reinforcement learning (RL) is the collection of all effective actions of the model, determining the range of action settings. The present work considers the buying and selling of stock in the stock market, thus only involving a discrete action space, including actions such as buying, selling, and wait-and-seeing. The proposed model uses a fully connected NN as the output layer to output a one-dimensional (1D) tensor containing three elements. First, the model uses the program to generate a random number subject to 0 and 1 uniform distributions. Suppose the random number is less than the preset value. In that case, the index corresponding to the largest element is selected as the action value among the three elements of the output vector. Otherwise, an integer in 0 and 2 is randomly selected as the action value. By referring to relevant codes and combined with multiple tests, the present experiment sets the preset value to 0.85. Action value = 1 indicates a stock-buying action. Similarly, action value = 2 suggests a stock-selling action. Finally, action value = 0 means a hold-and-waiting action.

**Figure 7:**
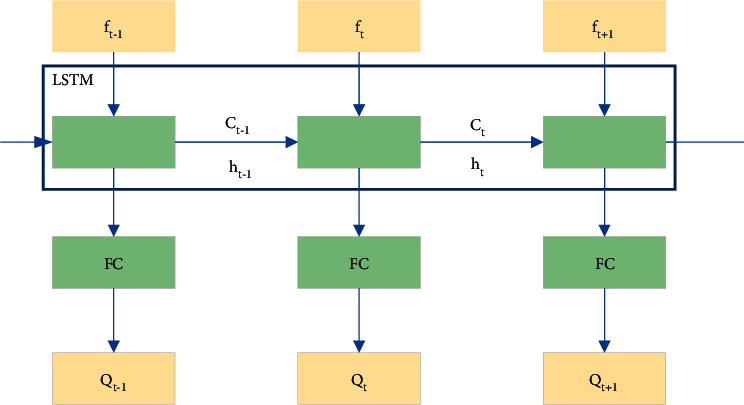
Transaction network structure based on LSTM.

Next, the proposed model is trained in the following ways: the LSTM NN is used to approximate the state function, namely, to estimate the *Q* value. The model input is the current time state *f*_*t*_, and the network outputs the estimated value *Q*(*s* and *a*) of all actions in the action space.  Figure 7 plots the specific network structure of approaching the *Q* value

In Figure 7, the *Q* value is estimated at each time, and the Mean Square Error (MSE) is taken as the loss function of the training model [30]. Then, the following equation calculates the MSE.

(5) $$\begin{array}{c} \text{MSE}=\frac{1}{N}\sum_{i=1}^{N}{\left(\left|y_{i}-{\hat{y}}_{i}\right|\right)}^{2}. \end{array}$$

In Figure 7, the real-time *Q* value is estimated to be *Q*(*s*, *a*), and the target *Q* value is expressed by *r* + *γ *max *Q*(*s*′, *a*′). Differently put, the target *Q* value equals the expected *Q* value after the state is transferred to s′+ the current time environmental reward *r*. *γ* is the attenuation factor ∈ [0, 1]. Afterward, the loss function should be minimized to maximize the *Q* value. After the gradient of the loss function is obtained, the parameters are updated iteratively. According to the GDM, the model loss function and parameters are renewed by equations (6)–(8):

(6) $$\begin{array}{c} Q^{*}\left(s,a\right)⟵Q\left(s,a\right)+\alpha \left\{r+\gamma \text{max}Q\left(s^{\prime },a^{\prime }\right)-Q\left(s,a\right)\right\}, \end{array}$$

(7) $$\begin{array}{c} L\left(s,\;a|\theta_{i}\right)={\left(r+\gamma \text{max}Q\left(s^{\prime },a^{\prime }|\theta_{i}\right)-Q\left(s,a\right)|\theta_{i}\right)}^{2}, \end{array}$$

(8) $$\begin{array}{c} \theta_{i+1}=\theta_{i}+\alpha \nabla_{\theta }L\left(\theta_{i}\right). \end{array}$$

In equations (6)–(8), *a* is the learning rate. When *Q* ≈ *Q*^*∗*^, *π*_*Q*_(*s*)=arg_*a*_max*Q*(*s*, *a*) can approximately represent the optimal strategy. According to equation (6), the predicted *Q* value and the target *Q* value use the same parameter model. When the predicted *Q* value increases, the target *Q* value also increases, which increases the risk of model fluctuation and divergence to a certain extent. Hence, to solve this problem, the DQN algorithm employs two different learning networks: the prediction network can evaluate the cost function of the current state-action pair, and the target network can generate the target *Q* value. Meanwhile, the loss function can update the parameters of the prediction network model. After multiple iterations, the parameters in the prediction network are copied to the parameters in the target network. Significantly, DQN introduces the target network to stabilize the target *Q* value for some time, reduces the correlation between the predicted *Q* value and the target *Q* value, and improves the algorithm's stability.

**Figure 8:**
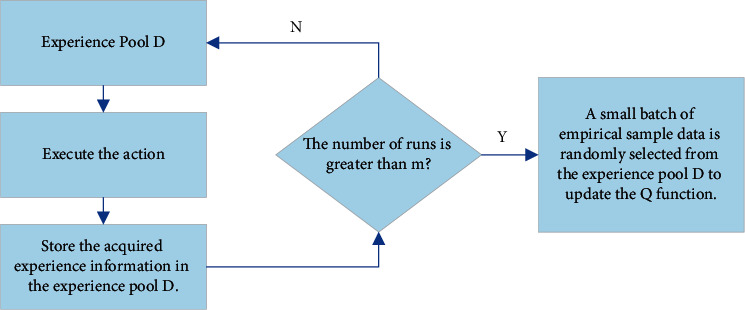
Experience playback mechanism.

The following experiment will use the ER mechanism for the DQN algorithm. First, it pools the experience sample data obtained through the agent-environment interaction at each time step (namely, the experience pool). Then, it randomly selects a small batch of data from the experience pool to train the network. DQN algorithm saves a certain amount of historical experience sample data. Each experience sample is stored in the form of (*s*, *a*, *r*, *s*′, and *T*) quintuple (indicating that the agent performs the action (*a*) in the state (*s*) and reaches the new state (*s′*) and obtains the corresponding reward *r*. Meanwhile, *T* is a Boolean value indicating whether the new state (*s′*) is terminated). Thus, the ER mechanism effectively removes the sample correlation and dependence so that the DNN model can better learn the cost function in RL tasks. The principle of the experience playback mechanism is given in Figure 8.

The specific process is as follows:

  Step 1: initialize experience pool *D* with a capacity of *n* to store training samples.
  Step 2: the state function Q is set as the prediction network, and the weight parameter *θ* is initialized randomly.
  Step 3: set the state value function *Q*′ as the target network and initialize the weight parameters *θ′* to *θ*.
  Step 4: set the maximum running times of the model as *m*.
  Step 5: randomly select the action *a*_*t*_ or calculate the action *Q*-value corresponding to the current state according to the network. Select the action with the largest *Q*-value as the optimal action.
  Step 6: the model performs the action (*a*_*t*_) to obtain the reward signal (*r*_*t*_) and state (*s*) from environmental feedback.
  Step 7: store the obtained state transition parameters into experience pool *D*.
  Step 8: the model randomly takes out the relevant information of calibration batch status from experience pool *D*.
  Step 9: calculate the target value of each state, and the model updates the *Q* value through the reward *r* after the target network *Q*′ performs the action.
  Step 10: update the weight parameter *θ* of the *Q* network based on small-batch samples using equations (6)–(8).
  Step 11: every time, after c iterations, update the network parameter *θ′* of the target action cost function *Q*′ as a parameter *θ* of the prediction network.

This section selects five stocks from the current Chinese A-shares, numbered A-E, to form an asset pool. Then, the initial 500,000 amount of the account is divided into five equal shares, and five stocks are invested and traded, respectively. Each stock is trained by indexes, including the opening price (open), closing price (close), highest price (high), lowest price (low), transaction volume (volume), MA10, MA21, RSI, momentum (MOM), and exponential moving average (EMA). Then, the open, close, high, and low are obtained through the Tushare financial data interface, while MA10, MA21, RSI, MOM, and EMA are obtained by calling TA-lib.

## 5. Experimental Results and Analysis

### 5.1. Experimental Result

**Figure 9:**
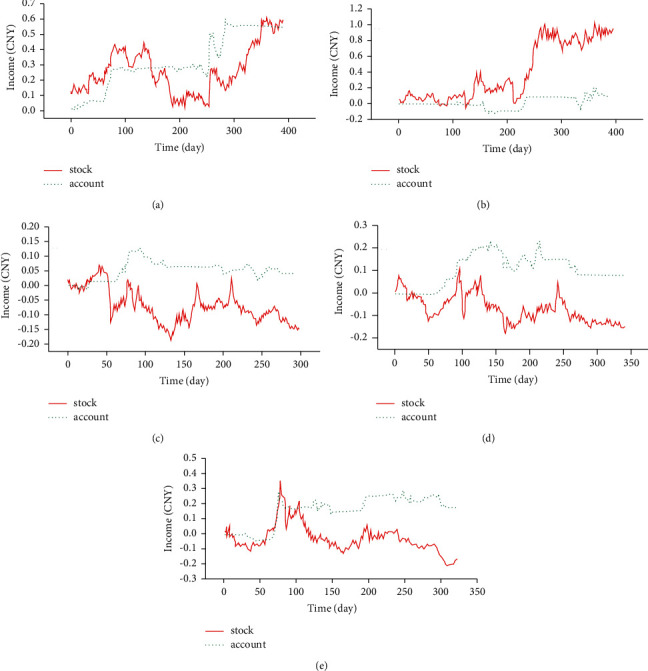
Trading behavior and account income of the (a-e) stock model.

Next, the five stocks' data are divided into two parts: the data from January 1, 2012, to May 29, 2019 (used for model training), and the data from May 30, 2019, to December 31, 2021 (used for back-test inspection).  Figure 9 charts the inspection results.

Figures 9(a)–9(e) display the trading behavior and account income of the A-E stock model, respectively.

**Figure 10:**
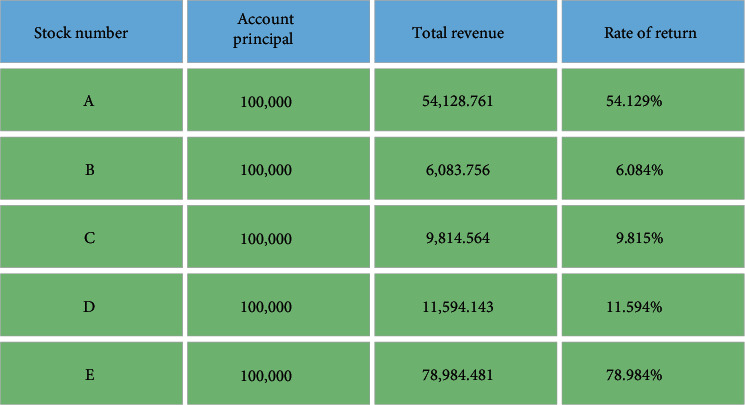
Model back-test results.

In Figure 9, the abscissa and the ordinate are the trading time and stock price increase. The red line indicates the stock price trend during the back-test, while the green line represents the trend of proxy account value during the back-test. The specific back-test results of each stock are summarized in Figure 10

In Figure 10, the total return is the account's market value on the last day of the back-test period minus the initial principal. The rate of return (RoR) is the percentage of the quotient of the total return and the account's principal. Overall, the RoR of the agent account during the back-test period is 32.12%, and the performance is relatively ideal. The Shanghai Stock Index (SSI) increase in the same period is 19.157%. Thus, the model's performance has exceeded the increase of the SSI in the same period. The five stocks all have achieved positive returns during the back-test period in terms of individual stocks. E stock achieved the highest yield, 78.984%, while the B stock has achieved the lowest, 6.084%.

### 5.2. Index Analysis

**Figure 11:**
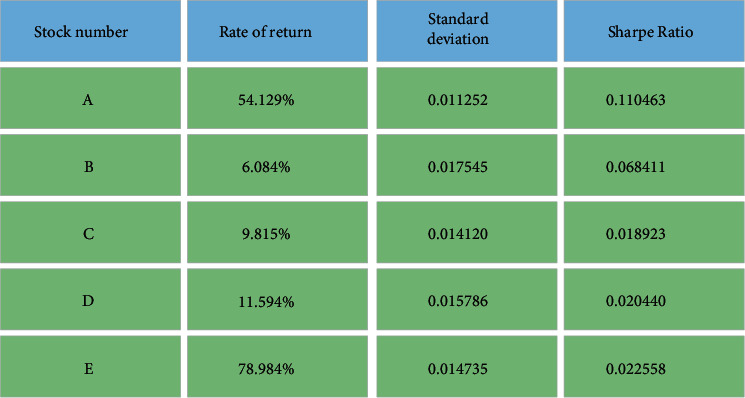
Comparison of SD and SR of each stock.

The back-test results are further analyzed by sharp ratio (SR). Specifically, the calculation of SR takes each trading day as the cycle. Generally speaking, the smaller the cycle is, the higher the calculation frequency is, and the greater the return volatility is. Since it is more difficult to make profits every trading day than every year, the calculation method with high calculation frequency can more comprehensively and genuinely reflect the risk-return status of account investment. The SD and SR of each stock are illuminated in Figure 11.

As corroborated by Figure 11, the SR of the five stocks in the account is positive, indicating that the average NGR of the stocks in the measurement period exceeded the risk-free interest rate. The larger the SR is, the higher the risk return is. Therefore, the proposed financial SIM model based on the DL algorithm is scientific and feasible.

## 6. Conclusions

At present, most trading strategies combined with DL focus on portfolio screening and rise and fall prediction. However, there are still few studies on reducing trading risk effectively. This paper mainly studies the financial SIM based on DL and obtains the excess return by exploring a new model. Firstly, the DQN algorithm is integrated into financial SIM, and then the model based on LSTM is constructed. Secondly, the model is trained by historical data, the training results are normalized, and GDM optimizes the model. Finally, the trained model is tested. As a result, five stocks of Chinese A-shares are selected to form an asset pool. The initial 500,000 amount of the account is divided into five equal shares, and the five stocks are invested and traded, respectively. The test results corroborate that the overall RoR of the model account during the back-test is 32.12%, and the maximum RoR falls on E stock with 78.984%. The minimum RoR goes to B stock with 6.084%, which is a positive return, and the SR of each stock is positive. Therefore, the proposed financial SIM model based on the DL algorithm is scientific and feasible. Regarding research limitations, this paper only used the basic stock market data and derived technical indicators without considering other factors. The lack of such data might determine the upper limit of the model. Later, other features affecting the stock price will be added to the data set to enhance the generalization and robustness of the model. The research content has certain reference significance for the DL-based financial SIM.

## Data Availability

The data used to support the findings of this study are included within the article.
